# CEACAM1 Is Associated With an Immune‐Activated Tumor Microenvironment and Therapeutic Stratification in Sarcoma

**DOI:** 10.1155/ijog/3351890

**Published:** 2026-04-30

**Authors:** Ziqi Cao, Minjue Shan, Yadong Guo, Mengmei Zhang, Ziyou Lin

**Affiliations:** ^1^ Department of Surgery, Heilongjiang University of Chinese Medicine Affiliated Second Hospital, Harbin, 150001, China; ^2^ School of Medicine, Tongji University, Shanghai, 200092, China, tongji.edu.cn; ^3^ Department of Urology, Shanghai Tenth People’s Hospital, School of Medicine, Tongji University, Shanghai, China, tongji.edu.cn; ^4^ Department of Clinical Laboratory, Shanghai Tenth People’s Hospital, Tongji University, Shanghai, China, tongji.edu.cn

**Keywords:** CEACAM1, immunotherapy response, sarcoma, tumor immune microenvironment

## Abstract

Carcinoembryonic antigen–related cell adhesion molecule 1 (CEACAM1) is a multifunctional immunomodulatory protein involved in both immune activation and immune suppression across diverse cancer types. However, its biological significance and clinical relevance in sarcoma (SARC) remain poorly defined. A comprehensive multiomics analysis of CEACAM1 was performed by integrating transcriptomic, genomic, epigenetic, immunological, and pharmacogenomic data from TCGA, GTEx, CPTAC, and large‐scale drug sensitivity datasets. CEACAM1 expression patterns, prognostic value, immune infiltration characteristics, pathway associations, genomic alterations, DNA methylation, transcriptional regulation, and therapeutic response were systematically evaluated. In SARC, elevated CEACAM1 expression was significantly associated with favorable clinical outcomes and characterized by an immune‐enriched tumor microenvironment. High CEACAM1 expression correlated with increased CD8^+^ T‐cell infiltration, enhanced interferon‐γ signaling, enrichment of tertiary lymphoid structure signatures, and upregulation of immune effector genes. Functional enrichment analyses further linked CEACAM1 to immune‐related pathways, including inflammatory response, interferon signaling, and apoptosis. At the therapeutic level, CEACAM1 expression was associated with reduced sensitivity to multiple chemotherapeutic agents, while concurrently correlating with enrichment of immunotherapy‐responsive gene signatures, indicating a distinct immune‐active yet therapeutically heterogeneous subtype of SARC. Collectively, these findings suggest that CEACAM1 represents a potential biomarker of immune activation and therapeutic stratification in SARC, warranting further investigation as a predictive indicator and immunotherapy‐related target in mesenchymal malignancies.

## 1. Introduction

Cancer remains a leading cause of morbidity and mortality worldwide, with diverse histological subtypes and complex tumor microenvironments posing significant challenges to effective treatment [1, 2]. Among them, sarcomas (SARC) are a heterogeneous group of malignant tumors originating from mesenchymal tissues, accounting for approximately 1% of adult and 15% of pediatric solid tumors [3, 4]. Despite their relative rarity, SARC are often aggressive, with high rates of metastasis, recurrence, and resistance to conventional therapies [5]. The immunologically “cold” nature of many SARC subtypes further limits the efficacy of immune checkpoint inhibitors, highlighting the urgent need to identify novel biomarkers and therapeutic targets to guide treatment and improve outcomes in this difficult‐to‐treat cancer [6, 7].

Carcinoembryonic antigen‐related cell adhesion molecule 1 (CEACAM1) is a member of the immunoglobulin superfamily that plays diverse roles in cell adhesion, immune modulation, angiogenesis, and tumor biology [8, 9]. Initially identified for its role in cell–cell interactions and immune homeostasis, CEACAM1 has since been implicated in both immune activation and immune suppression depending on cellular context and disease state [10, 11]. In recent years, accumulating evidence has highlighted its importance in cancer immunity, where it modulates T‐cell activity, natural killer (NK) cell inhibition, and cytokine signaling [12]. CEACAM1 has been reported to exhibit heterogeneous expression patterns across human cancers, with tumor‐type–specific implications for prognosis and therapy response [13]. In solid tumors such as lung and melanoma, CEACAM1 has been shown to participate in immune evasion by interacting with inhibitory receptors on effector cells, thereby impairing antitumor immunity [14, 15].

Despite its known immunoregulatory functions, the pan‐cancer landscape of CEACAM1 expression, genomic regulation, immunological relevance, and clinical value remains insufficiently understood. In particular, the role of CEACAM1 in SARC—a highly heterogeneous group of mesenchymal malignancies with limited treatment options and poor immunotherapy response—has yet to be comprehensively elucidated. Whether CEACAM1 serves as a biomarker of immune activation or suppression in SARC, and whether it predicts therapeutic response, remains unclear.

In this study, we performed a comprehensive multiomics analysis of CEACAM1 across cancers with a focus on SARC. By integrating transcriptomic, genomic, epigenetic, immunological, and pharmacogenomic data from TCGA, GTEx, GDSC, and public immune databases, we investigated the expression profile, prognostic significance, regulatory mechanisms, and immunotherapeutic implications of CEACAM1. Such integrative multiomics approaches have been widely adopted in cancer research to uncover immune‐related biomarkers and therapeutic targets, providing a powerful framework for elucidating the complex tumor–immune landscape [16, 17]. Notably, by jointly capturing molecular alterations, immune contexture, and treatment‐related features, multiomics strategies enable a more comprehensive evaluation of immune‐associated biomarkers than single‐layer analyses, particularly in immunologically heterogeneous tumors such as SARC [18, 19]. Our findings provide novel insights into the context‐dependent functions of CEACAM1 and highlight its potential as an immune‐associated biomarker and therapeutic target in SARC and beyond.

## 2. Materials and Methods

### 2.1. Data Acquisition and Preprocessing

RNA sequencing data, clinical information, and survival outcomes for 33 cancer types were obtained from The Cancer Genome Atlas (TCGA) database using the UCSC Xena platform (https://xenabrowser.net/). Normal tissue data were supplemented with GTEx (Genotype‐Tissue Expression) datasets for improved differential expression analysis. Protein‐level expression data were retrieved from the Clinical Proteomic Tumor Analysis Consortium (CPTAC). All expression values were normalized as transcripts per million (TPM) and log2‐transformed for downstream analyses. SARC was selected as the cancer of interest for in‐depth analysis.

### 2.2. Differential Expression and Survival Analysis

Differential expression of CEACAM1 between tumor and normal tissues across cancer types was assessed using the Wilcoxon rank‐sum test. Kaplan–Meier survival analysis was performed to evaluate the prognostic significance of CEACAM1 with respect to overall survival (OS), disease‐specific survival (DSS), progression‐free interval (PFI), and disease‐free interval (DFI). Hazard ratios (HRs) and corresponding *p* values were calculated using univariate Cox proportional hazards regression models. Restricted cubic spline models were applied to explore potential nonlinear associations between CEACAM1 expression and clinical outcomes.

### 2.3. Immune Infiltration and Immune Subtype Analysis

Immune cell infiltration levels were estimated using multiple well‐established immune deconvolution algorithms. Tumor microenvironment features, such as immune and stromal scores, were calculated using the multiple established computational methods. These complementary approaches were applied to comprehensively characterize immune cell composition and stromal content within tumor samples.

In addition, pan‐cancer immune subtype classification was performed according to the framework established by Thorsson [19], which defined six immune subtypes (C1–C6) based on immune cell infiltration patterns, immune gene expression signatures, tumor heterogeneity, and clinical outcomes across TCGA cancers.

### 2.4. Functional Enrichment Analysis

Gene set variation analysis (GSVA) was performed to identify differentially enriched pathways in CEACAM1 high‐versus low‐expression groups using hallmark gene sets. KEGG pathway analysis was conducted using the R package. Gene set enrichment analysis (GSEA) was further used to identify immune‐related and oncogenic pathways associated with CEACAM1.

### 2.5. Genomic Alteration and Epigenetic Regulation

Copy number alteration (CNA) data were obtained from the TCGA using GISTIC2.0 output. Correlations between CEACAM1 expression and CNAs, as well as genome instability (FGA/FGL/FGG), were computed. Methylation beta values were retrieved from the Illumina Human Methylation 450K dataset and compared between tumor and normal tissues. Site‐specific methylation effects on CEACAM1 were visualized and analyzed. Potential transcription factor regulators of CEACAM1 were identified using the Cistrome database.

### 2.6. Immunogenomic Correlations and Immune Checkpoint Analysis

Associations between CEACAM1 expression and immune checkpoint genes, chemokines, chemokine receptors, and major histocompatibility complex (MHC) molecules were systematically evaluated. Immune‐related activities, including T cell–inflamed signatures, IFN‐γ response, and chemokine expression, were evaluated using predefined gene sets. Enrichment scores were calculated, and differences between CEACAM1 expression groups were compared. In addition, relationships between CEACAM1 expression and somatic mutation–derived neoantigen load were assessed using standard computational pipelines.

### 2.7. Drug Sensitivity Analysis

Drug response data were obtained from the Genomics of Drug Sensitivity in Cancer (GDSC1/2), Cancer Therapeutics Response Portal (CTRP), and PRISM repurposing datasets. Spearman correlation analysis was used to evaluate associations between CEACAM1 expression and drug sensitivity metrics, including IC50 and area under the dose–response curve (AUC). Compounds with statistically significant associations were visualized for further interpretation.

### 2.8. Tumor Immune Cycle and Tertiary Lymphoid Structure (TLS) Estimation

The seven‐step cancer–immunity cycle was quantified using single‐sample gene set enrichment analysis (ssGSEA), as previously described by Chen et al. CEACAM1 expression was correlated with each step of the immune cycle to assess its involvement in anti‐tumor immune processes. TLS scores were estimated using validated TLS‐associated gene signatures (e.g., CXCL13, CCL19) and ssGSEA‐based scoring methods.

### 2.9. Statistical Analysis

All statistical analyses were conducted in R software (Version 4.2.1). The Wilcoxon rank‐sum test, Spearman correlation, Cox regression, and chi‐square test were used as appropriate. *p* values < 0.05 were considered statistically significant. Visualization was performed using ggplot2, ComplexHeatmap, and survminer.

## 3. Results

### 3.1. CEACAM1 Exhibits Differential Expression Across Cancer Types and Is Associated With Prognosis

To assess the expression profile of CEACAM1 in cancer, we performed a pan‐cancer analysis using TCGA and GTEx datasets. As shown in Figure 1(a), CEACAM1 was significantly downregulated in tumor tissues compared to normal tissues in multiple cancer types, including BRCA, CHOL, COAD, HNSC, and KIRC (*p* < 0.05). Integration of TCGA and GTEx data further confirmed these findings, demonstrating consistent downregulation of CEACAM1 in tumors across most cancer types (Figure 1(b)). Additional validation using independent datasets from the CPTAC cohort revealed similar trends at the protein level in selected cancers (Figure 1(c)). We next investigated the prognostic significance of CEACAM1 expression using univariate Cox regression analysis. High CEACAM1 expression was associated with good OS in several cancers, (Figure 1(d)), and was also linked to better DSS (Figure 1(e)). For PFI, high CEACAM1 expression predicted good outcomes in certain cancer types (Figure 1(f)). These findings suggest that CEACAM1 may serve as a potential prognostic biomarker in specific cancer types.

FIGURE 1Differential expression and prognostic value of CEACAM1 across cancers. (a) CEACAM1 expression in tumor versus normal tissues across TCGA cancer types. (b) Pan‐cancer expression analysis integrating TCGA and GTEx datasets. (c) Protein‐level validation of CEACAM1 expression using CPTAC datasets. (d–f) Univariate Cox regression analysis of CEACAM1 expression for overall survival (OS), disease‐specific survival (DSS), and progression‐free interval (PFI).

**Figure:**
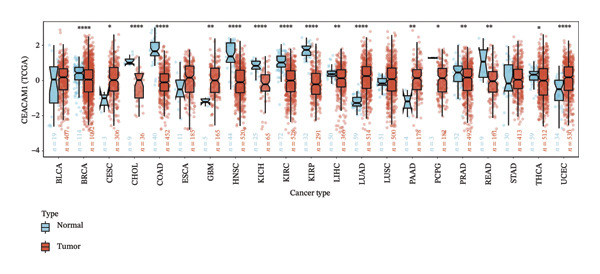
(a)

**Figure:**
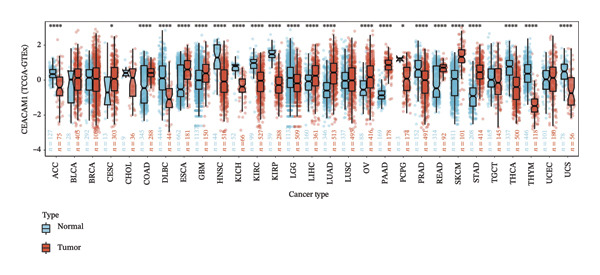
(b)

**Figure:**
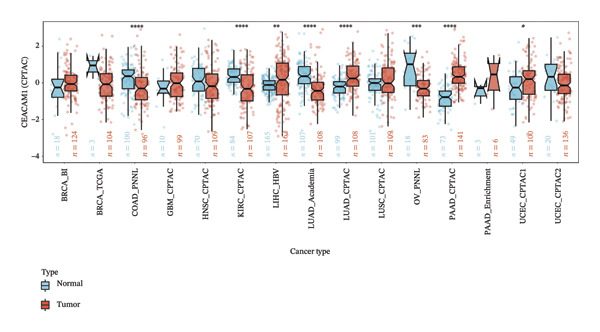
(c)

**Figure:**
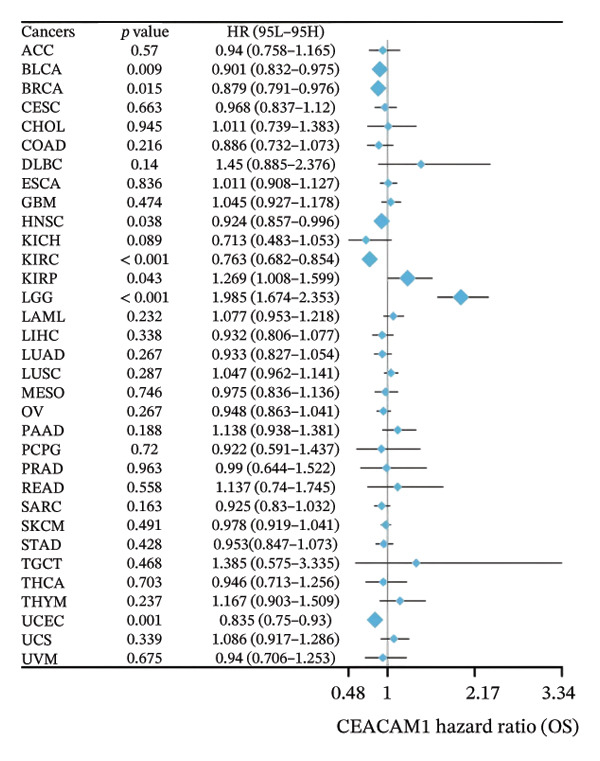
(d)

**Figure:**
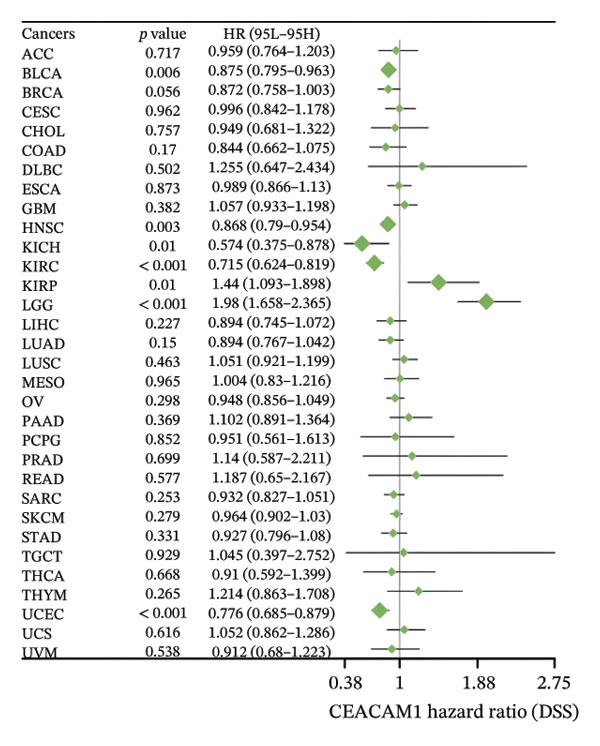
(e)

**Figure:**
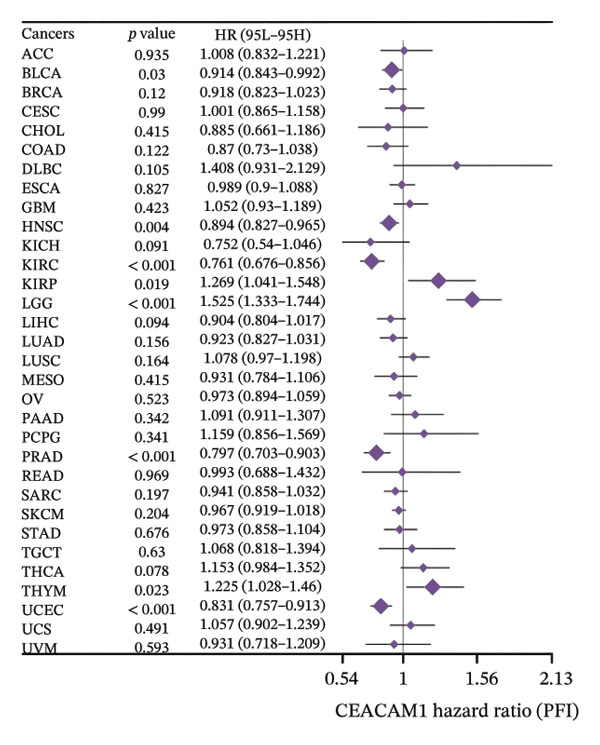
(f)

### 3.2. CEACAM1‐Related Genomic Alterations and Immunological Characteristics Across Cancers

To explore the molecular and immunological relevance of CEACAM1 across cancers, we performed a comprehensive multidimensional analysis. A heatmap summarizing CEACAM1 expression and its correlation with immune cells across a broad range of datasets is shown (Figure 2(a)). Further immune infiltration analysis showed that CEACAM1 expression was significantly correlated with a wide spectrum of immune cell infiltration estimates (Figure 2(b)). We also analyzed the relationship between CEACAM1 expression and hallmark cancer pathways. CEACAM1 was positively associated with pathways such as angiogenesis, inflammation and quiescence, but negatively correlated with cell cycle and DNA repair pathways, indicating its dual roles in tumor immunity and suppression of proliferative programs (Figure 2(c)). Analysis of genomic alterations using multiple datasets revealed that CEACAM1 had a low alteration frequency overall, with relatively higher frequencies observed in endometrial, ovarian epithelial, and melanoma tumors (Figure 2(d)). We next assessed the association between CEACAM1 expression and SNV‐derived neoantigen burden across cancer types. The radar plot showed weak to moderate positive correlations in certain tumors, suggesting that CEACAM1 may reflect tumor immunogenicity in specific contexts (Figure 2(e)). To better understand the immunological landscape associated with CEACAM1, we stratified 9,073 TCGA patients into six pan‐cancer immune subtypes. High CEACAM1 expression was significantly enriched in C2 (IFN‐γ dominant) and C1 (wound healing) subtypes (Figure 2(f)). CEACAM1 showed good diagnostic accuracy (AUC > 0.6) in cancers such as CHOL, LIHC, and BRCA in both TCGA and TCGA–GTEx cohorts, supporting its potential as a diagnostic biomarker (Figure 2(g)).

FIGURE 2Multidimensional immunological and genomic characterization of CEACAM1 across cancers. (a) Heatmap of CEACAM1 expression and immune cell correlations. (b) Correlation between CEACAM1 and immune cell infiltration by subtype. (c) Association with hallmark cancer pathways. (d) Frequency and types of CEACAM1 genomic alterations. (e) Correlation with SNV‐derived neoantigen burden. (f) CEACAM1 expression enriched in immune subtypes. (g) ROC curves demonstrating the diagnostic potential of CEACAM1 across cancers.

**Figure:**
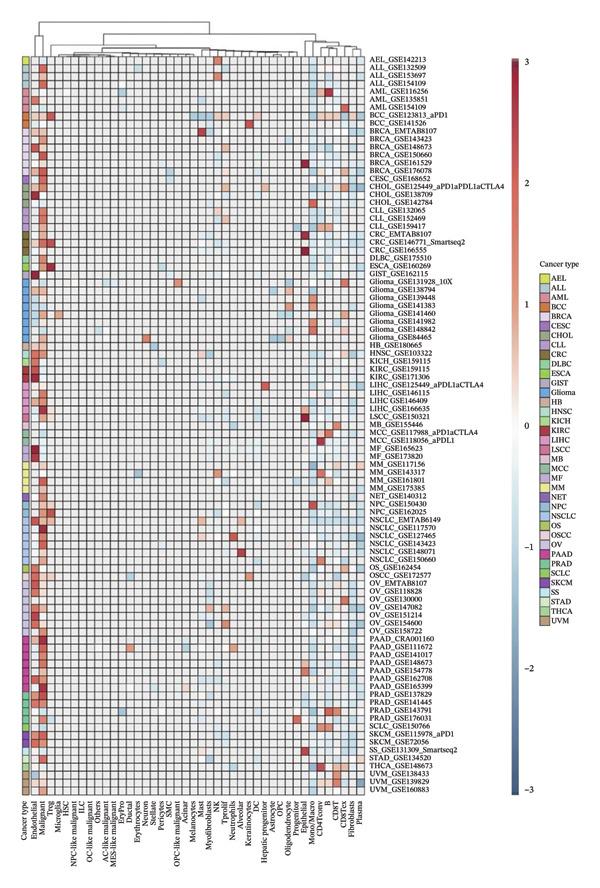
(a)

**Figure:**
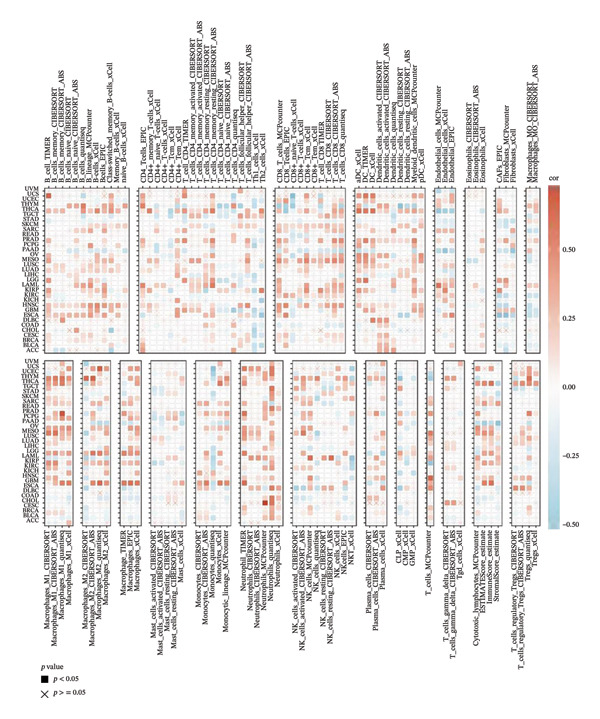
(b)

**Figure:**
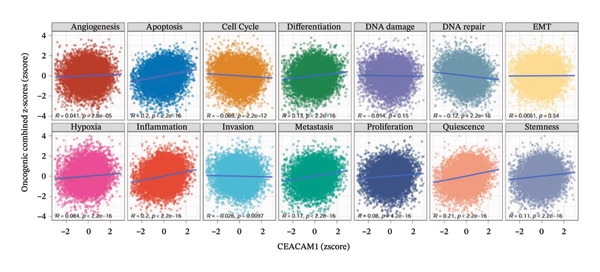
(c)

**Figure:**
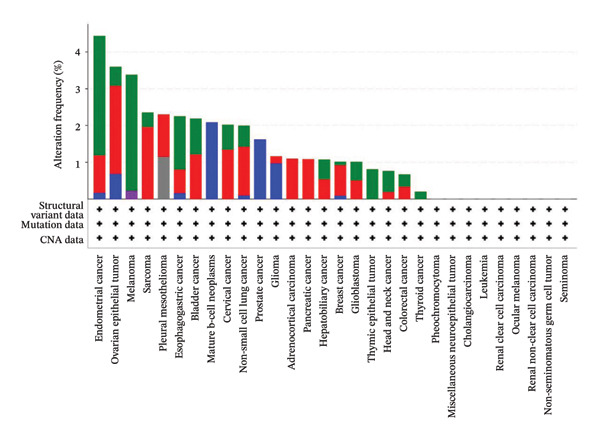
(d)

**Figure:**
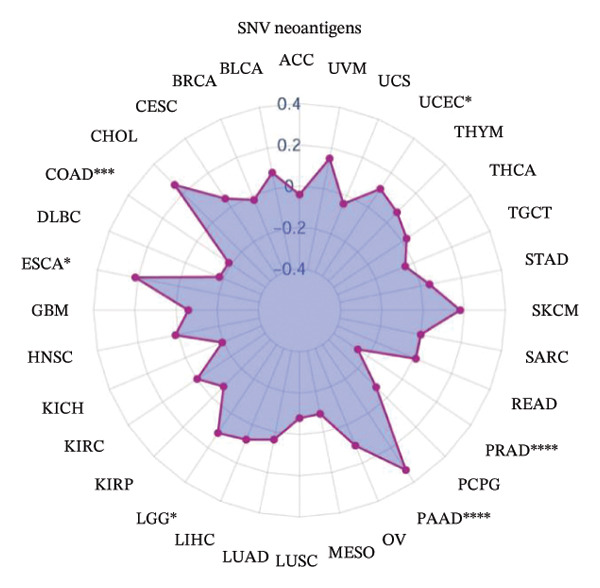
(e)

**Figure:**
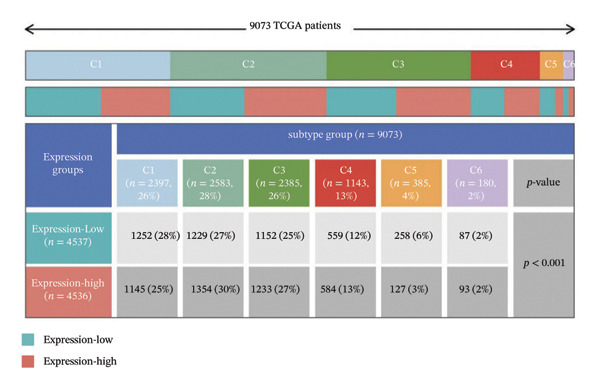
(f)

**Figure:**
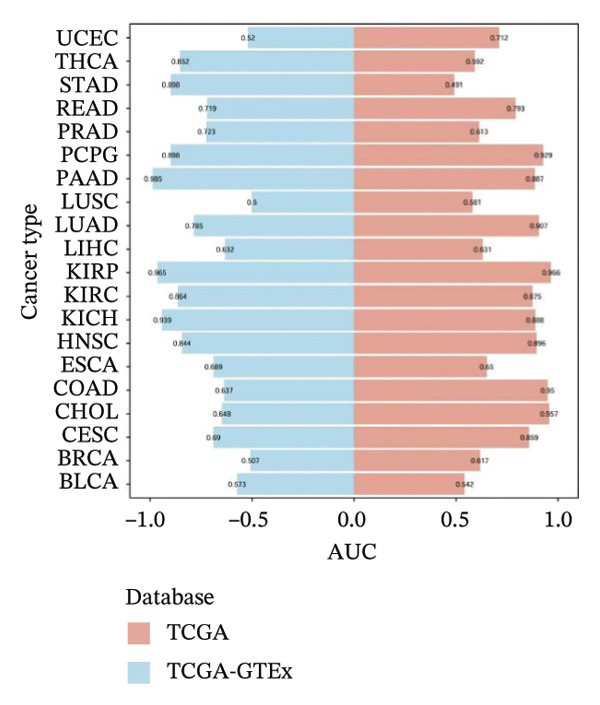
(g)

### 3.3. CEACAM1 Expression Is Associated With Prognosis and Immunological Interactions in SARC

Kaplan–Meier survival analysis demonstrated that high CEACAM1 expression was significantly associated with better clinical outcomes in SARC patients, including OS (*p* = 0.002; Figure 3(a)), DSS (*p* = 0.009; Figure 3(b)), PFI (*p* = 0.012; Figure 3(c)), and DFI (*p* = 0.029; Figure 3(d)). Restricted cubic spline analysis was performed to explore the relationship between CEACAM1 expression and survival outcomes in SARC (Figures 3(e), 3(f), 3(g), 3(h)). To explore the immunological context of CEACAM1, we conducted a joint analysis with CD8+ T‐cell infiltration scores. A scatter plot stratified patients into four subgroups based on high/low CEACAM1 and CD8 expression, revealing distinct immune phenotypes (Figure 3(i)). Subsequent survival analysis showed no significant difference (Figures 3(j), 3(k)). Chi‐square analysis showed no significant difference in the survival status across CEACAM1 expression quartiles in TCGA–SARC (*p* = 0.075). In GSE17119, the progression status differed across quartiles (*p* = 0.011) (Figures 3(l), 3(m)).

FIGURE 3Prognostic and immunological significance of CEACAM1 in SARC. (a–d) Kaplan–Meier analysis shows high CEACAM1 expression predicts favorable OS, DSS, PFI, and DFI. (e–h) Restricted cubic spline curves of CEACAM1 expression in relation to survival outcomes. (i–k) Distribution and survival analyses of patients stratified by CEACAM1 expression and CD8+ T‐cell infiltration levels. (l–m) Distribution of outcome status across CEACAM1 expression quartiles in the TCGA‐SARC and GSE17119 cohorts.

**Figure:**
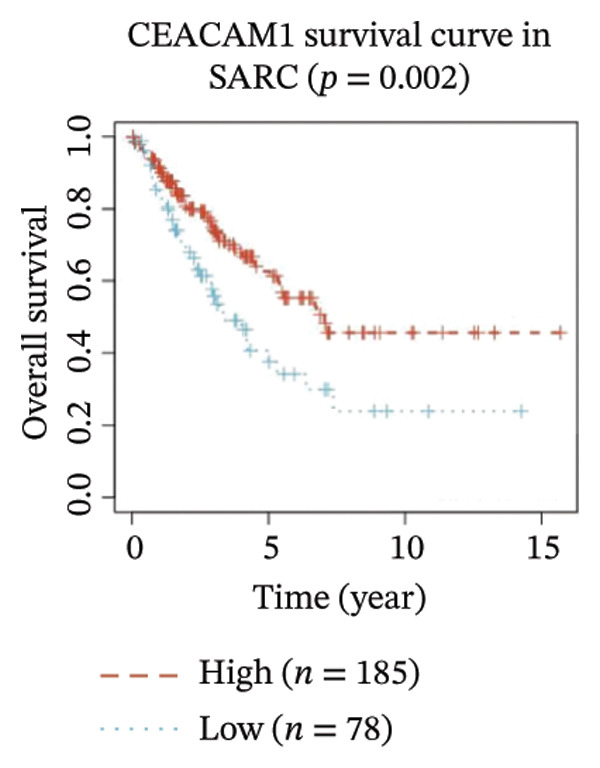
(a)

**Figure:**
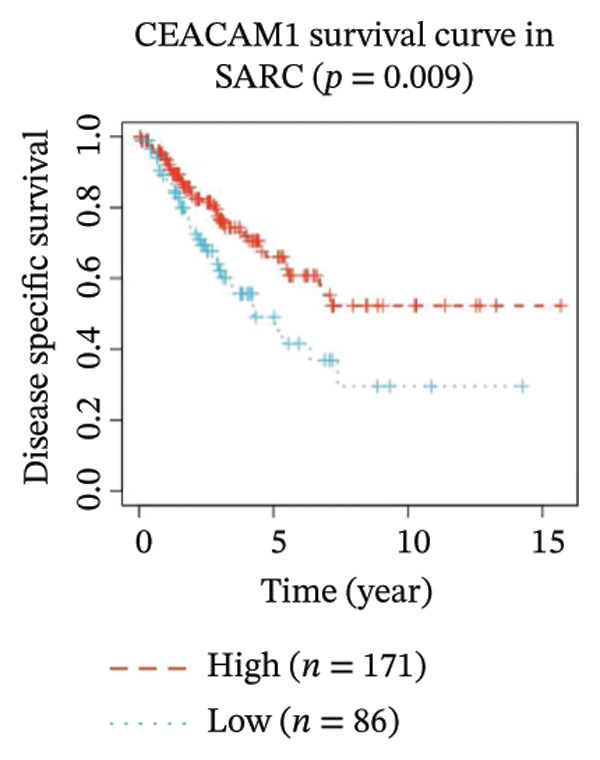
(b)

**Figure:**
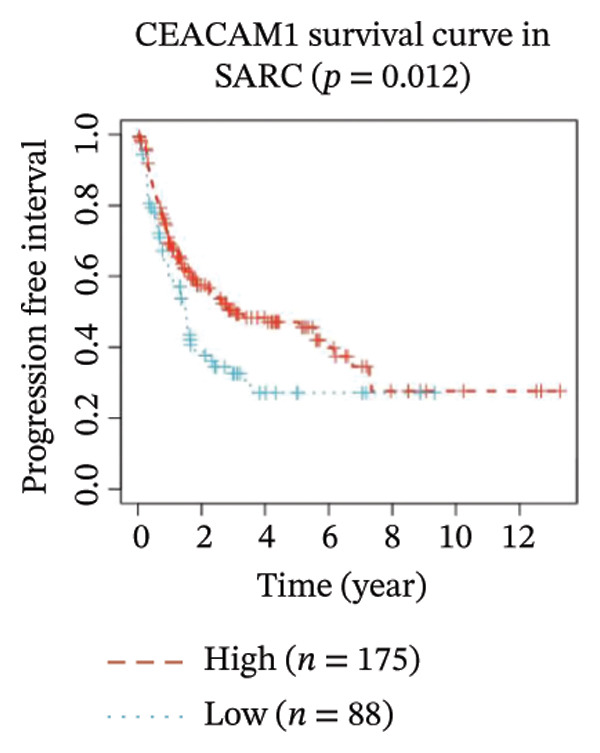
(c)

**Figure:**
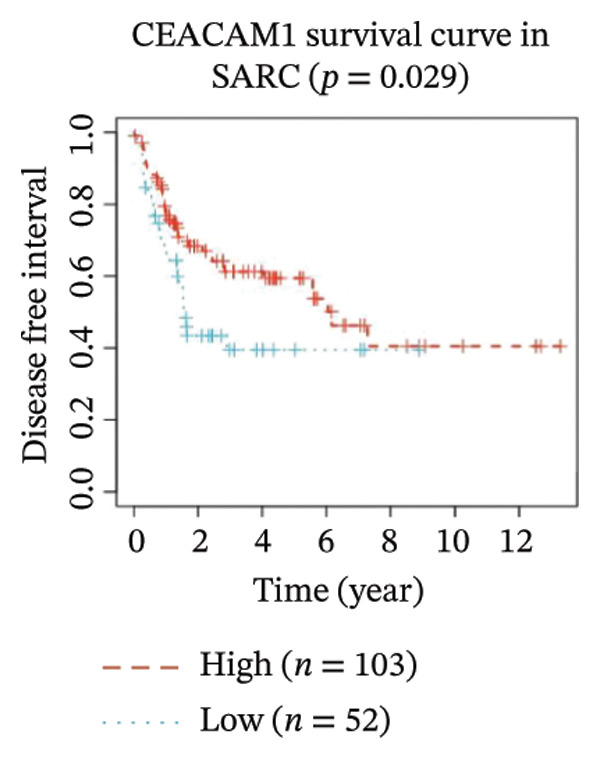
(d)

**Figure:**
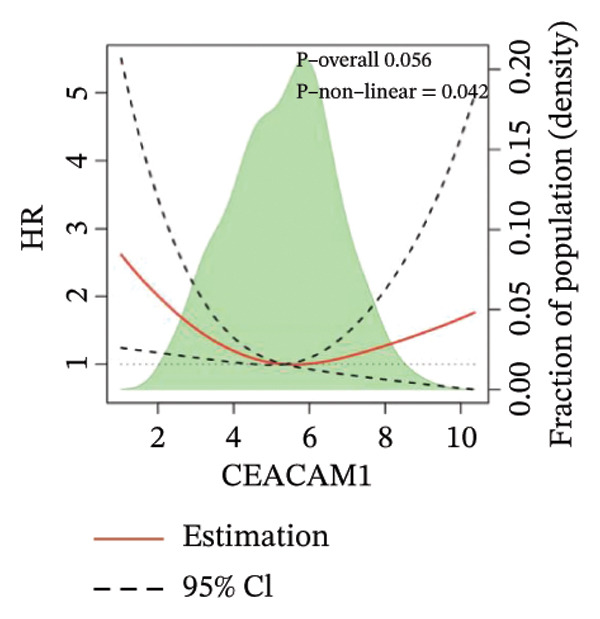
(e)

**Figure:**
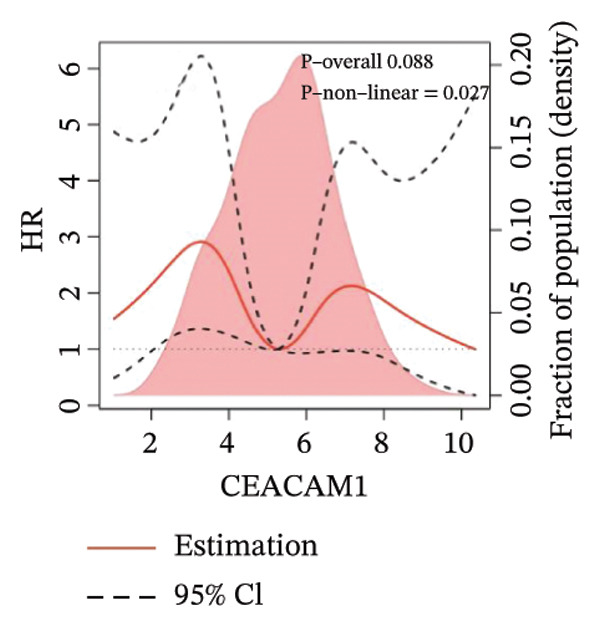
(f)

**Figure:**
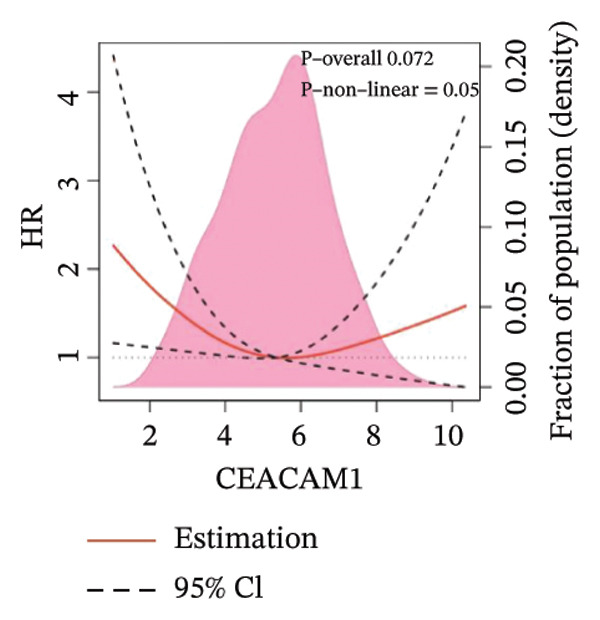
(g)

**Figure:**
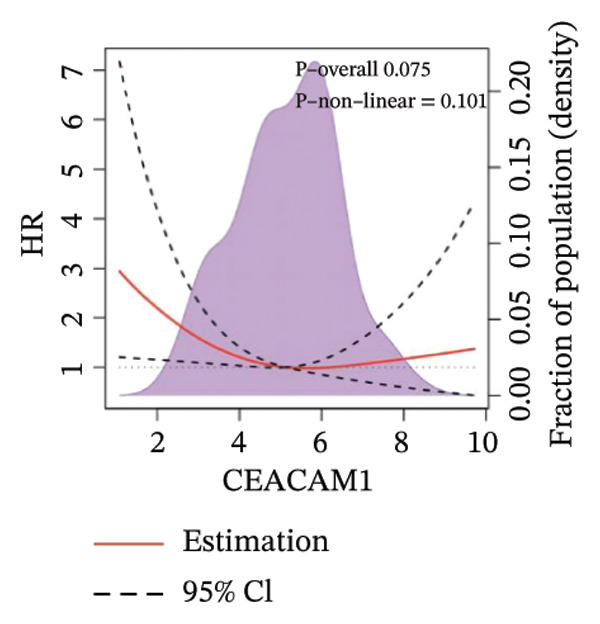
(h)

**Figure:**
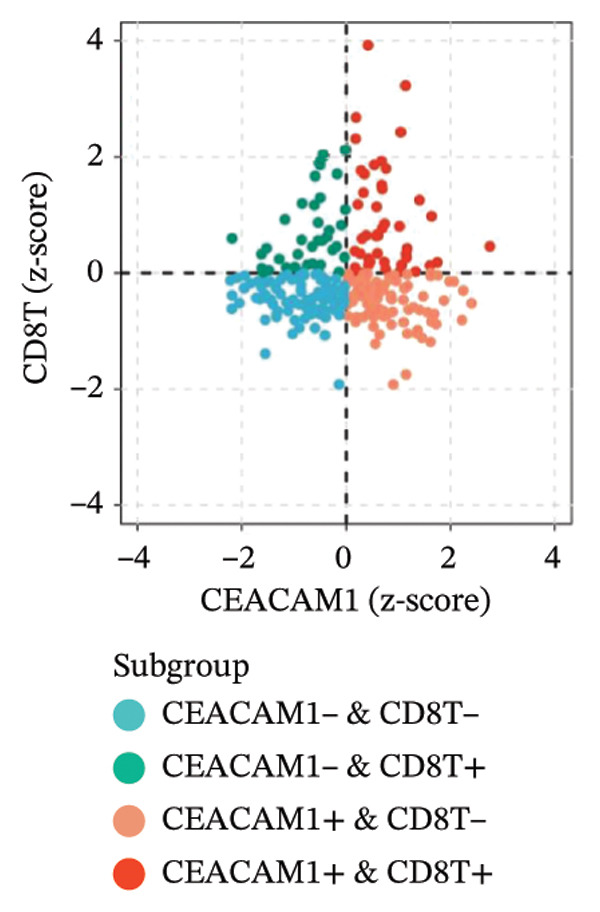
(i)

**Figure:**
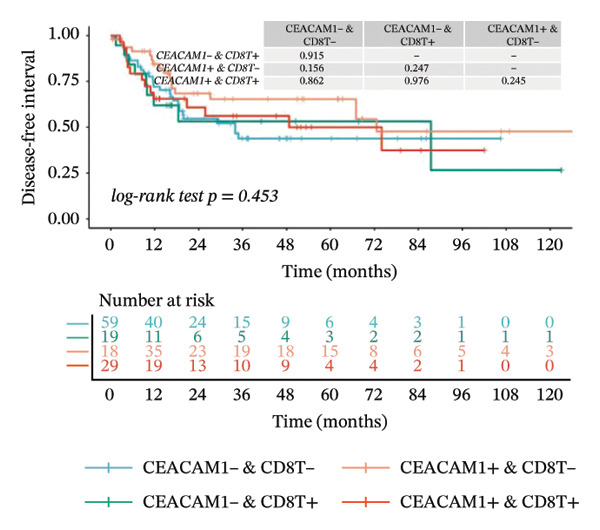
(j)

**Figure:**
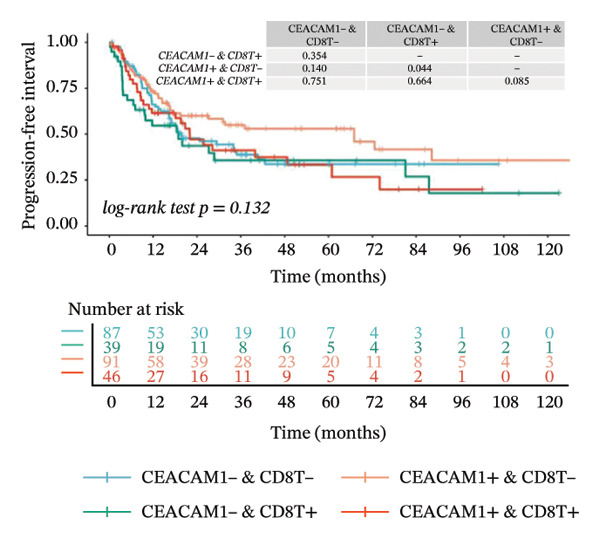
(k)

**Figure:**
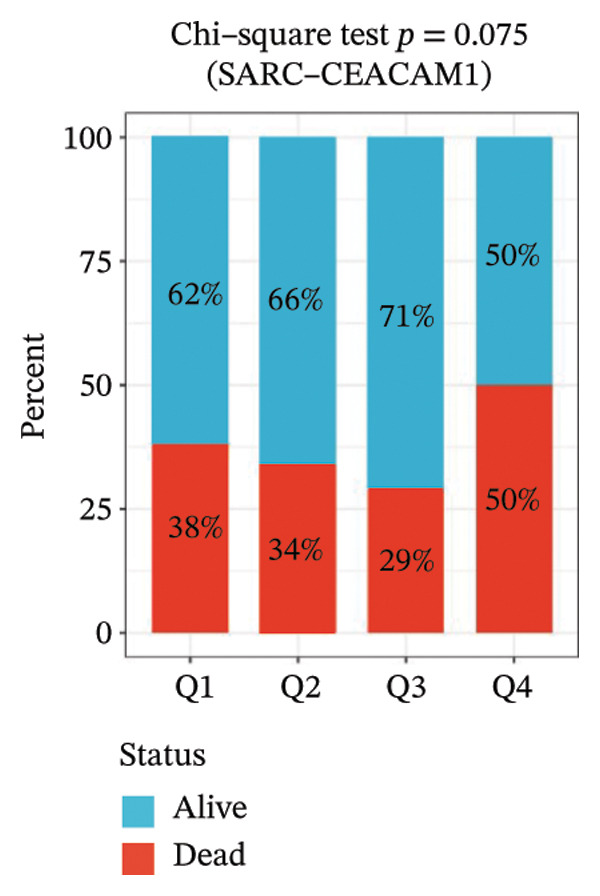
(l)

**Figure:**
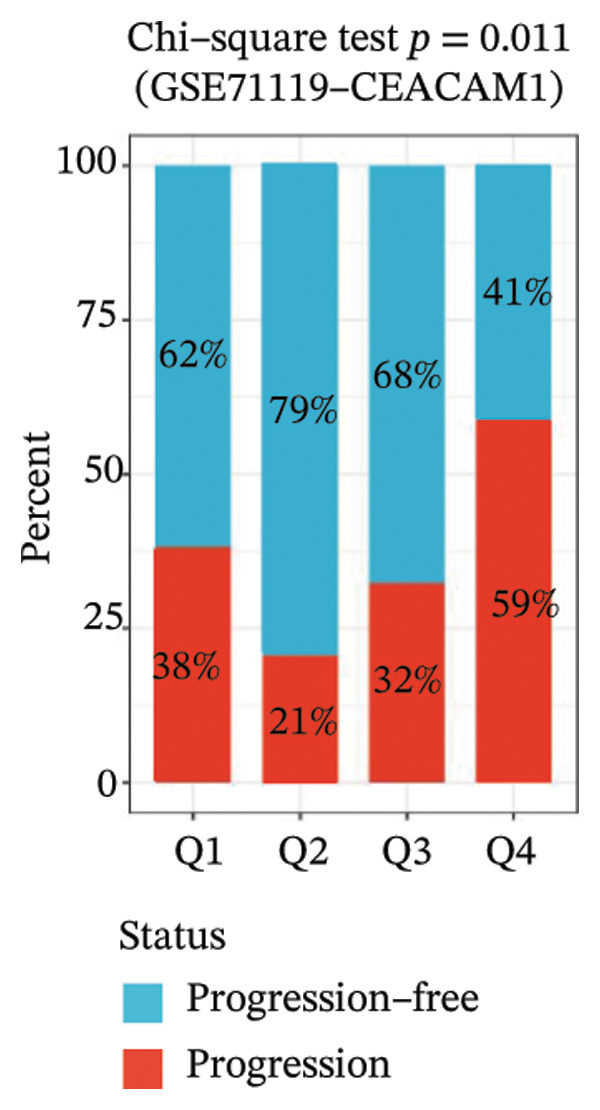
(m)

### 3.4. Functional Characterization of CEACAM1 in SARC Reveals Associations With Proliferation, Immune Regulation, and Oncogenic Pathways

To elucidate the functional roles of CEACAM1 in SARC, we first analyzed its correlation with hallmark oncogenic processes. CEACAM1 expression was significantly negatively correlated with cell cycle (*R* = −0.19, *p* = 0.00021) and DNA repair (*R* = −0.18, *p* = 0.0036), while showing positive correlations with inflammatory response (*R* = 0.21, p = 0.00074), proliferation (*R* = 0.23, *p* = 0.00016), and stemness (*R* = 0.24, *p* = 0.00011) (Figure 4(a)). GSVA‐based pathway enrichment analysis showed that CEACAM1 high expression was associated mainly with the enrichment of metabolic pathways, especially lipid and amino acid metabolism pathways (Figure 4(b)). Further KEGG enrichment analysis confirmed these findings, showing that CEACAM1 was involved in multiple cancer‐related pathways, including cell growth and signal transduction (Figure 4(c)). To visualize pathway‐level interactions, we constructed a correlation chord diagram that revealed strong associations between CEACAM1 and immune and cancer‐related signaling modules, including the PI3K/Akt, mTOR, and apoptosis pathways (Figure 4(d)). These correlations highlight CEACAM1’s potential role as a regulator of both tumor cell survival and immune signaling in the microenvironment.

FIGURE 4Functional and pathway analysis of CEACAM1 in SARC. (a) Correlation analysis between CEACAM1 expression and hallmark oncogenic processes. (b) Gene set variation analysis (GSVA) comparing high and low CEACAM1 expression groups. (c) KEGG pathway enrichment analysis based on CEACAM1‐associated genes. (d) Chord diagram showing correlations between CEACAM1 and selected immune and signaling pathways.

**Figure:**
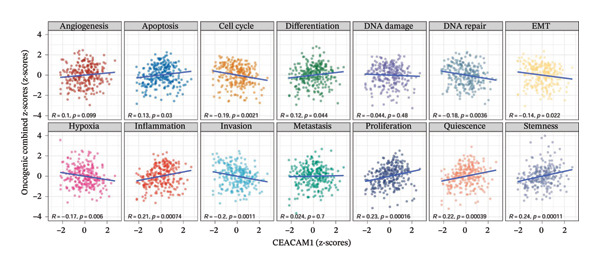
(a)

**Figure:**
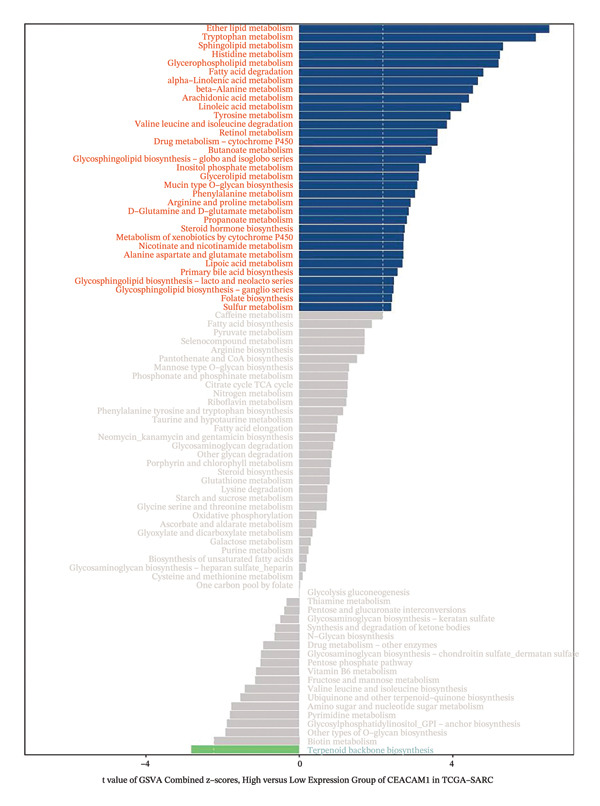
(b)

**Figure:**
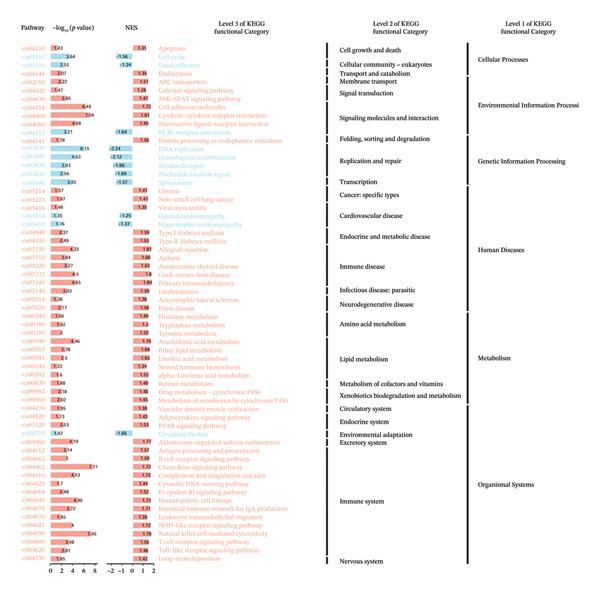
(c)

**Figure:**
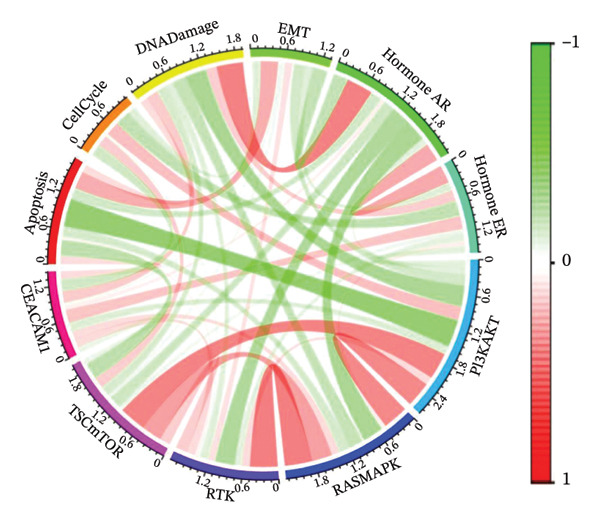
(d)

### 3.5. CEACAM1 Is Associated With Immunosuppressive Microenvironment Features and Immune Checkpoint Regulation

To investigate the immunological role of CEACAM1 in SARC, we analyzed its relationship with various immune components. High CEACAM1 expression was positively correlated with the expression of multiple immunoinhibitory genes, chemokines, chemokine receptors, and HLA molecules (Figure 5(a)). CEACAM1 was associated with differential expression patterns of multiple co‐inhibitory and co‐stimulatory immune checkpoint genes across expression quartiles (Figure 5(b)). MeTIL analysis indicated that tumors with high CEACAM1 expression showed significantly elevated MeTIL scores (Figure 5(c)). Additionally, CEACAM1 expression was positively correlated with tumor microenvironment scores (*R* = 0.34 and *p* = 3e–08), suggesting its association with an immune‐enriched microenvironment (Figure 5(d)). A broader immune‐genomic correlation analysis demonstrated that CEACAM1 expression was positively associated with proliferation and intratumor heterogeneity, while negatively correlated with SNV neoantigens and the number of segments (Figure 5(e)). Correlation heatmaps across TCGA and GEO datasets confirmed that CEACAM1 expression was positively linked to CD8^+^ T cells and neutrophils, while negatively correlated with M0 macrophages (Figure 5(f)). Heatmap analysis showed that the high CEACAM1 expression group exhibited generally increased immune cell infiltration and higher stromal and microenvironment scores across multiple algorithms (Figure 5(g)). Collectively, these findings suggest that CEACAM1 contributes to an immune‐activated microenvironment in SARC.

FIGURE 5Immune‐related analyses of CEACAM1 expression in SARC. (a) Correlation analysis between CEACAM1 expression and immune checkpoint molecules, chemokines, chemokine receptors, and HLA genes. (b) Comparison of immune‐related gene copy number alterations and expression levels across CEACAM1 expression quartiles. (c) Distribution of MeTIL scores in CEACAM1 high and low expression groups. (d) Correlation between CEACAM1 expression and tumor microenvironment score. (e) Association of CEACAM1 expression with immune‐related signatures, genomic features, and TCR/BCR diversity across expression quartiles. (f) Correlation analysis between CEACAM1 expression and immune cell subset abundance. (g) Heatmap of immune cell infiltration and tumor microenvironment scores across CEACAM1 high and low expression groups.

**Figure:**
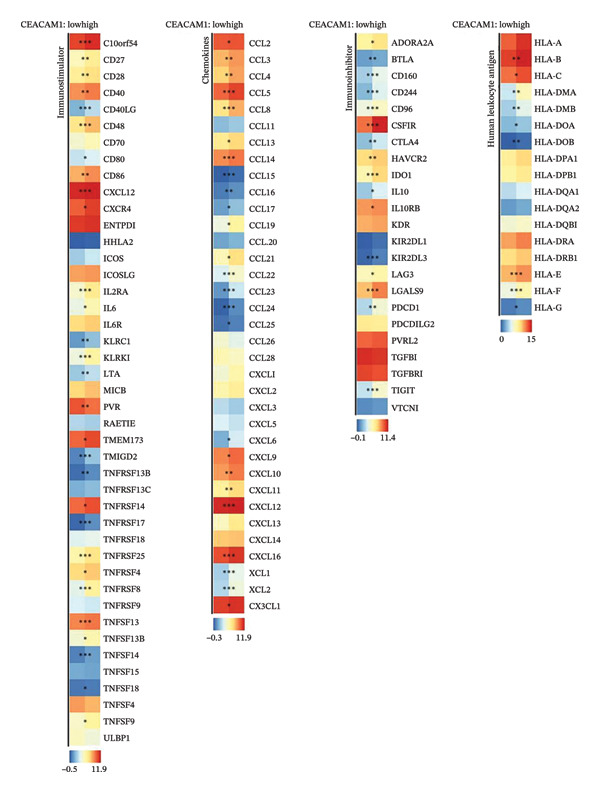
(a)

**Figure:**
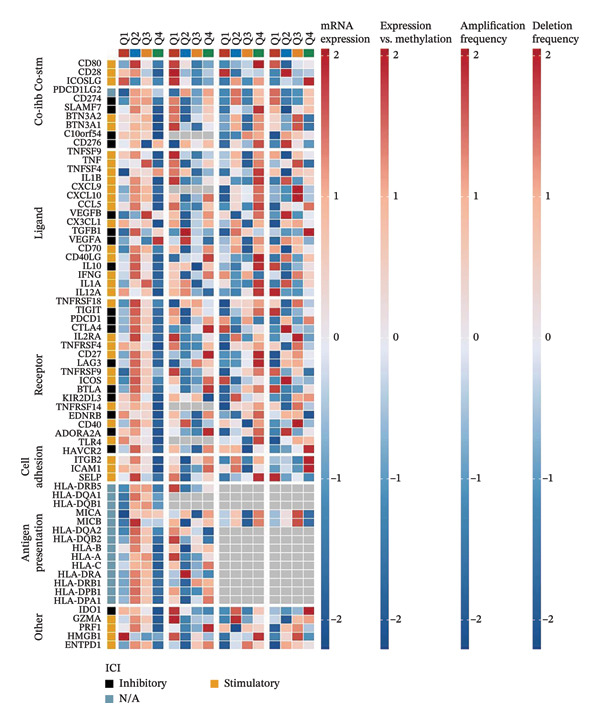
(b)

**Figure:**
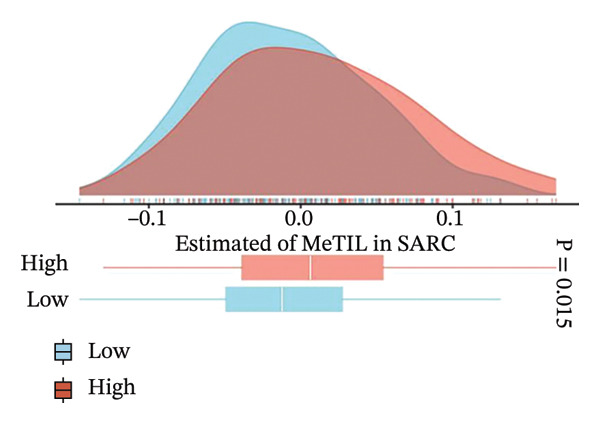
(c)

**Figure:**
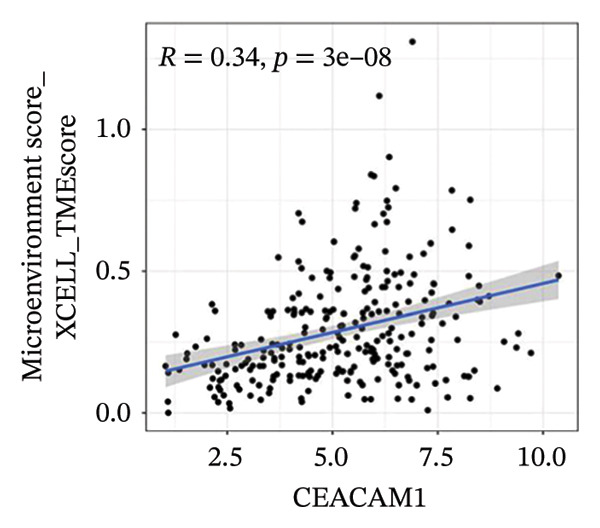
(d)

**Figure:**
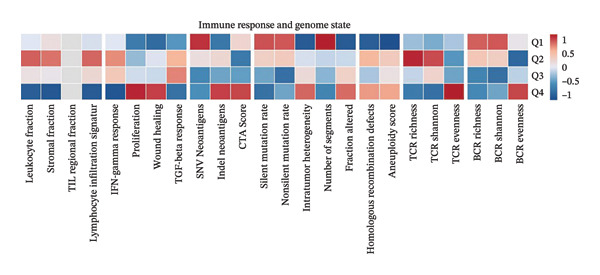
(e)

**Figure:**
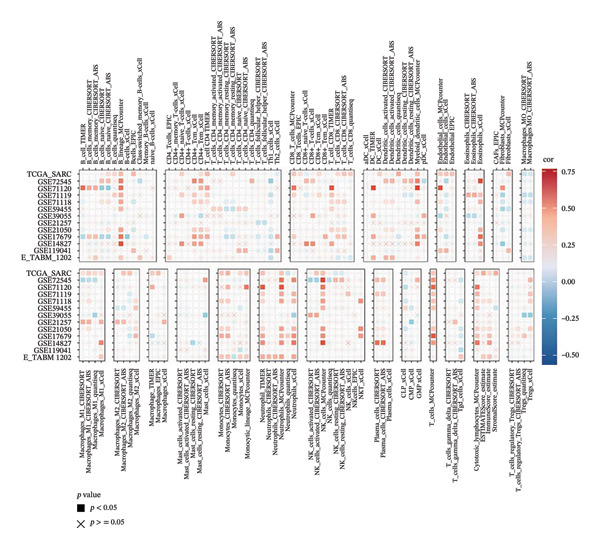
(f)

**Figure:**
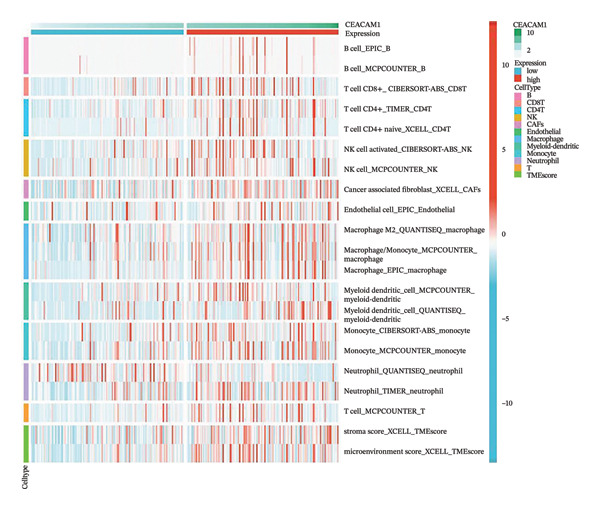
(g)

### 3.6. Genomic and Epigenetic Regulation of CEACAM1 in SARC

To explore the regulatory mechanisms underlying CEACAM1 expression in SARC, we first analyzed its CNAs (Figure 6(a)). Correlation analysis confirmed that CEACAM1 expression was not significantly associated with its copy number status in SARC (Figure 6(b)). We further assessed the relationship between CEACAM1 expression levels and the fraction of genome altered (FGA). Higher CEACAM1 expression quartiles (Q3–Q4) were associated with increased genomic instability (Figure 6(c)), suggesting CEACAM1 may be linked to broader genomic alterations. To evaluate transcriptional regulation, we utilized the Cistrome database to identify transcription factors potentially binding to the CEACAM1 promoter region. Multiple immune‐ and cancer‐related transcription factors were identified, indicating their likely involvement in CEACAM1 transcriptional activation (Figure 6(d)). Next, we assessed the epigenetic regulation of CEACAM1 via promoter methylation. No significant difference was observed in overall CEACAM1 promoter methylation between tumor and normal samples in SARC (Figure 6(e)). However, methylation levels varied considerably across individual CpG sites, with some sites (e.g., cg20657383 and cg14904363) exhibiting notably different methylation distributions (Figure 6(f)), suggesting site‐specific methylation may influence CEACAM1 expression heterogeneity. These results suggest that CEACAM1 expression in SARC is not driven by copy number changes or global promoter methylation but may be regulated by specific transcription factors and localized epigenetic modifications.

FIGURE 6Genomic and epigenetic features of CEACAM1 in SARC. (a) Overview of CEACAM1 copy number alterations. (b) Correlation between CEACAM1 expression and copy number variation. (c) Comparison of genome alteration fractions (FGA, FGG, and FGL) across CEACAM1 expression quartiles. (d) Prediction of transcription factors binding to the CEACAM1 promoter region using the Cistrome database. (e) Comparison of CEACAM1 promoter methylation levels between tumor and normal tissues. (f) Site‐specific CpG methylation profiling of the CEACAM1 promoter region.

**Figure:**
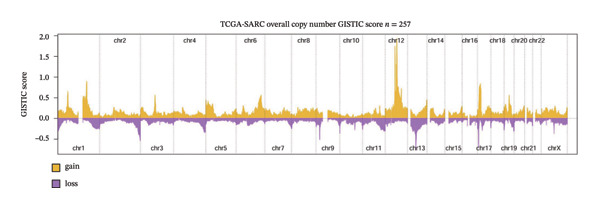
(a)

**Figure:**
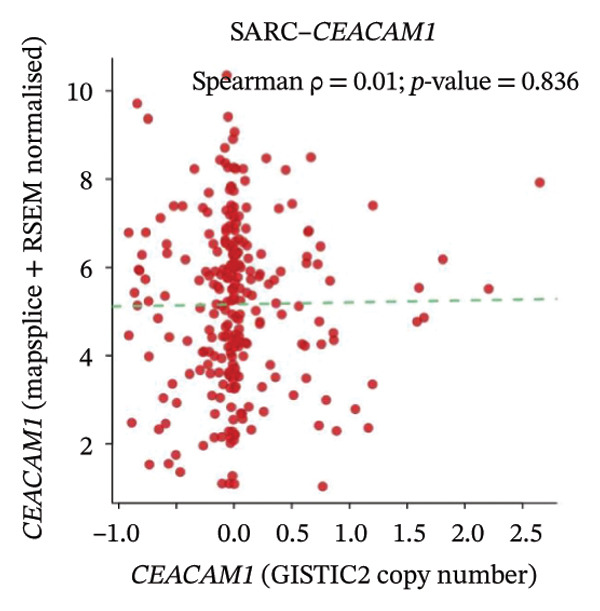
(b)

**Figure:**
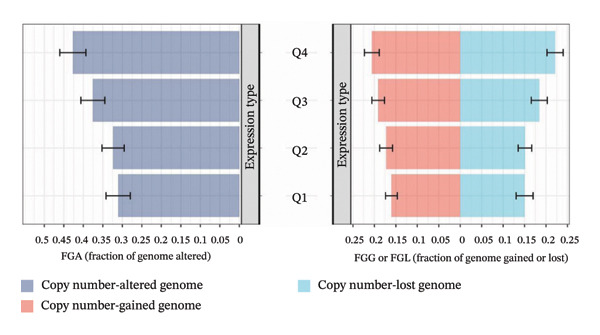
(c)

**Figure:**
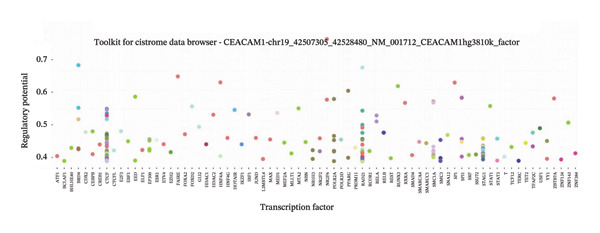
(d)

**Figure:**
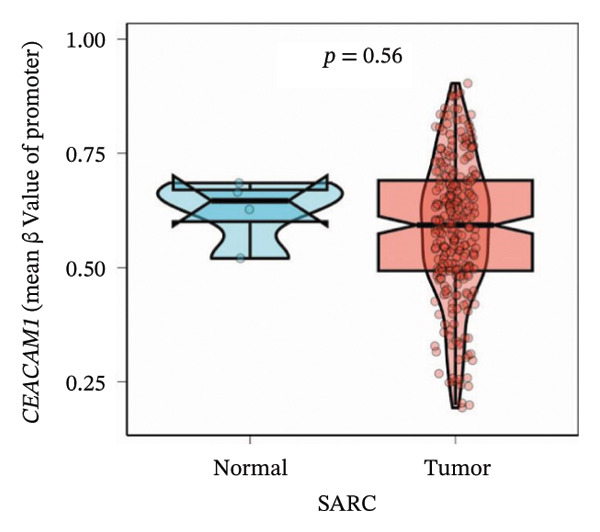
(e)

**Figure:**
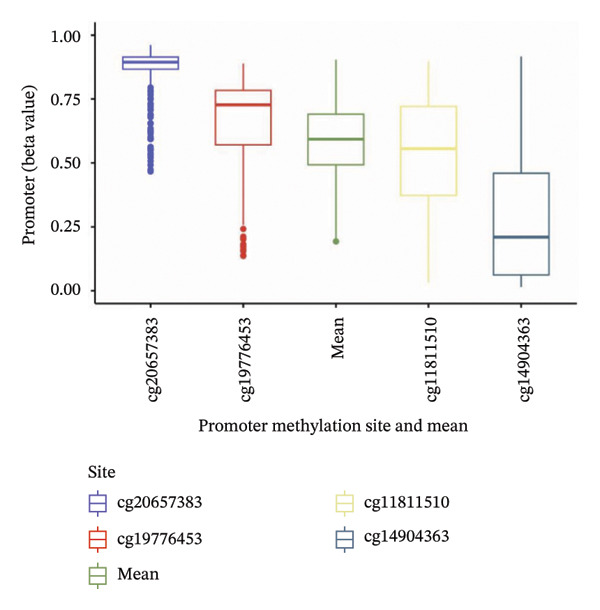
(f)

### 3.7. CEACAM1 Expression Correlates With Drug Sensitivity and Immune Activation Signatures in SARC

We analyzed its association with drug response across multiple pharmacogenomic databases. CEACAM1 expression was significantly correlated with sensitivity or resistance to various compounds in PRISM, CTRP, and GDSC datasets. Notably, CEACAM1 expression was positively associated with reduced sensitivity to chemotherapeutics such as cisplatin, docetaxel, and kinase inhibitors (Figure 7(a)). Stratified analysis revealed consistent associations between CEACAM1 expression and drug IC50/AUC values across GDSC1 (Figure 7(b)), PRISM (Figure 7(c)), and CTRP (Figure 7(d)), indicating its potential as a biomarker for drug response. To further understand the immunological landscape of CEACAM1, we evaluated its association with key immune activation markers. High CEACAM1 expression was significantly associated with expression of the T cell–inflamed gene signature (Figure 7(e)), IFN‐γ response genes (Figure 7(f)), and chemokine expression (Figure 7(g)), indicating a more inflamed and immunoactive tumor microenvironment. We then assessed CEACAM1’s involvement in the cancer–immunity cycle. CEACAM1 expression showed positive correlations with multiple steps, further supporting its role in facilitating antitumor immunity (Figure 7(h)). Additionally, CEACAM1 expression was significantly associated with TLS signatures (Figure 7(i)), which have been linked to enhanced immune responses and improved immunotherapy efficacy. CEACAM1 is not only linked to specific drug sensitivities but also serves as a marker of immune activation and enhanced antitumor immune processes in SARC.

FIGURE 7Drug response and immune activation signature analyses associated with CEACAM1 in SARC. (a–d) Association between CEACAM1 expression and drug sensitivity profiles in PRISM, CTRP, and GDSC datasets. (e) Correlation analysis between CEACAM1 expression and T cell–inflamed gene signature. (f) Correlation between CEACAM1 expression and IFN‐γ response–related genes. (g) Association with chemokine gene expression. (h) Analysis of CEACAM1 expression in relation to different steps of the cancer–immunity cycle. (i) Correlation between CEACAM1 expression and TLS‐associated gene signatures.

**Figure:**
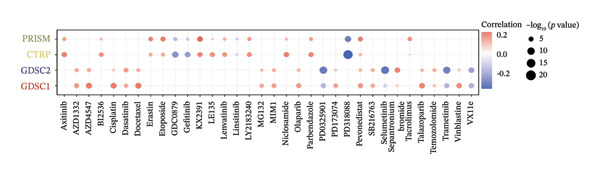
(a)

**Figure:**
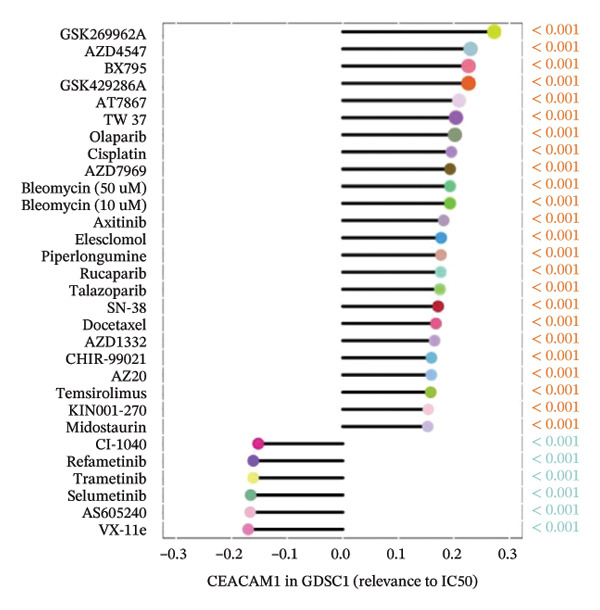
(b)

**Figure:**
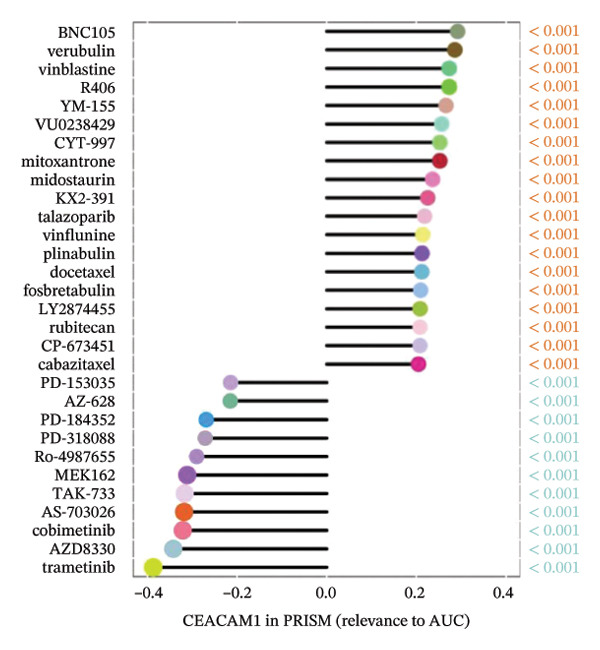
(c)

**Figure:**
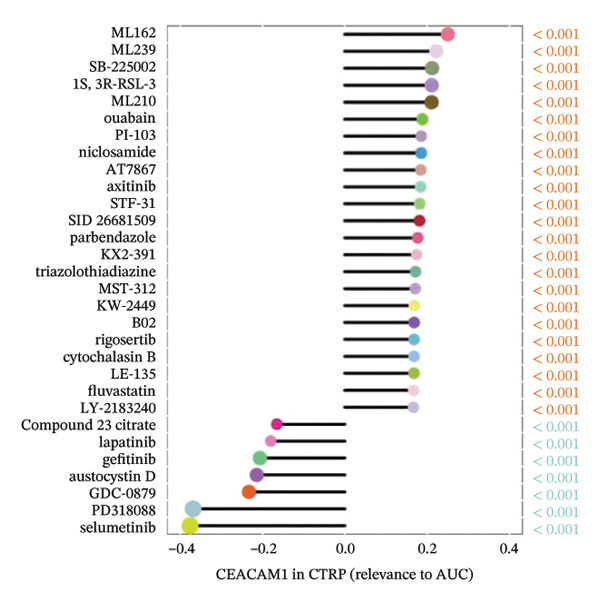
(d)

**Figure:**
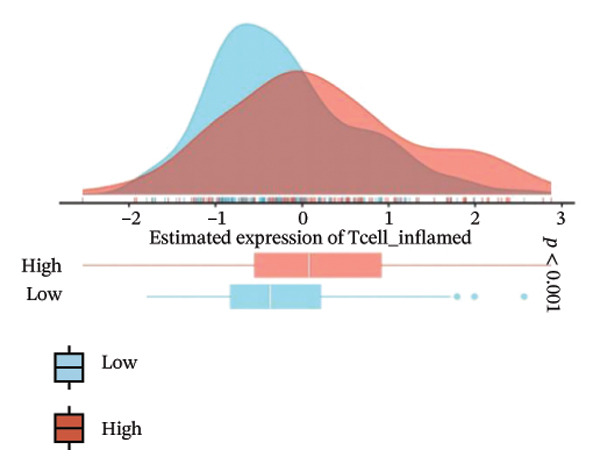
(e)

**Figure:**
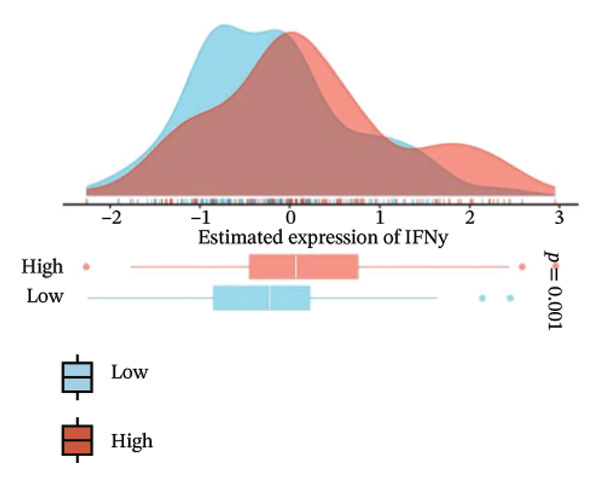
(f)

**Figure:**
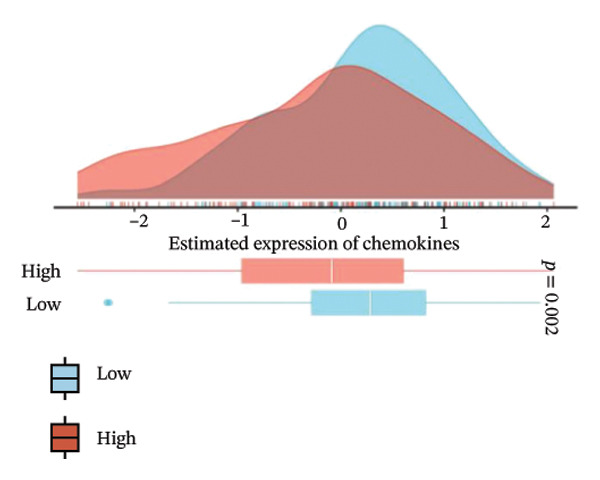
(g)

**Figure:**
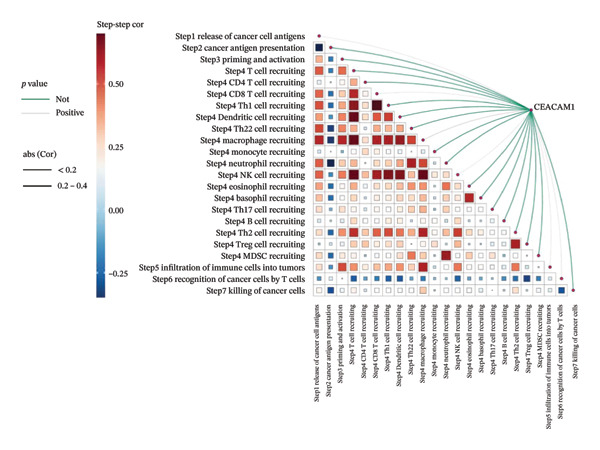
(h)

**Figure:**
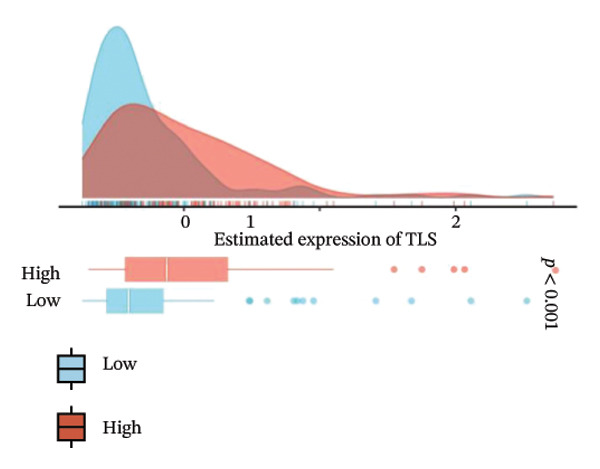
(i)

## 4. Discussion

In this study, we conducted a comprehensive multiomics analysis of CEACAM1 across human cancers, with a particular focus on SARC. Our results revealed that CEACAM1 is differentially expressed across cancer types and is significantly associated with favorable prognosis in SARC. These findings highlight CEACAM1 as a potential immune‐related biomarker with diagnostic and prognostic value in mesenchymal tumors.

CEACAM1 demonstrated strong associations with key immunological features in SARC. High expression of CEACAM1 was positively correlated with CD8^+^ T‐cell infiltration, immune effector gene signatures, interferon‐γ response, and TLS formation, suggesting its involvement in shaping an immune‐activated tumor microenvironment. These features have been linked to enhanced antitumor immunity indicating that CEACAM1 may reflect a functionally active immune contexture in SARC [21–23]. Additionally, CEACAM1 expression was significantly correlated with a variety of immune‐related genes, including MHC molecules, chemokines, and immune checkpoints. Interestingly, CEACAM1 was also associated with elevated immune inflitration and stemness scores, suggesting that its expression may mark an inflamed yet dynamically regulated immune state. CEACAM1 may therefore capture a complex immune contexture characterized by simultaneous immune activation and adaptive immune regulation, which could be amenable to therapeutic modulation or combination immunotherapy strategies in SARC [24]. Mechanistically, we found that CEACAM1 expression was not significantly driven by copy number variation or global promoter methylation. Instead, transcription factor prediction analysis pointed to immune‐regulatory factors such as STAT1 and SPI1 as potential upstream regulators of CEACAM1, supporting a model in which CEACAM1 is transcriptionally activated under inflammatory or immune‐stimulatory conditions. To contextualize CEACAM1 relative to established immune biomarkers, PD‐L1 expression reflects checkpoint signaling, TMB represents neoantigen load, and TLS indicates the spatial organization of adaptive immunity. In contrast, CEACAM1 appears to capture a broader immune contexture, integrating immune infiltration, interferon signaling, and immune–tumor interactions, supporting its complementary rather than redundant value in immune stratification.

From a therapeutic perspective, pharmacogenomic analysis revealed that CEACAM1 expression was correlated with altered sensitivity to a broad spectrum of compounds, including reduced sensitivity to traditional cytotoxic agents such as cisplatin and docetaxel. In contrast, its high expression was consistently associated with a T cell–inflamed phenotype and robust interferon signaling. Together, these findings suggest that CEACAM1 may have practical utility as an immune stratification marker, potentially complementing established biomarkers such as PD‐L1 expression, TMB, and TLS status, rather than serving as a stand‐alone predictor. In this context, CEACAM1 may assist in identifying SARC patients more likely to benefit from immunotherapy‐based strategies as opposed to conventional chemotherapy, thereby informing personalized treatment selection.

Emerging studies in melanoma, lung cancer, and gastrointestinal malignancies have shown that CEACAM1 can modulate T‐cell exhaustion and influence the efficacy of immune checkpoint blockade therapy [24]. Some clinical efforts have explored CEACAM1‐targeted antibodies or CEACAM1/PD‐1 dual blockade strategies to enhance antitumor immunity [25, 26]. Our findings, showing that CEACAM1 is linked to an immune‐active microenvironment and favorable prognosis in SARC, provide a rationale to explore whether these strategies may also be applicable in mesenchymal tumors. Importantly, CEACAM1 is readily detectable in clinical specimens using immunohistochemistry or transcript‐based assays, and its spatial distribution could be further interrogated through digital pathology or spatial transcriptomic platforms, supporting its feasibility as a translational biomarker without additional experimental burden. Moreover, the association of CEACAM1 with TLS formation, a known predictor of immunotherapy efficacy, further reinforces its potential as a translational biomarker for patient stratification and response prediction [26, 27].

Nonetheless, several limitations should be acknowledged. As with most computational biomarker studies, our analyses are primarily correlative and do not establish causality. In addition, reliance on bulk transcriptomic data limits the resolution of cell‐type–specific CEACAM1 expression. However, these limitations are partially mitigated by the use of multiple independent cohorts and the integration of diverse omics layers, which together enhance the robustness and consistency of our findings. Future studies involving retrospective clinical cohorts, immunotherapy‐treated populations, and integration with digital pathology pipelines will be important next steps to further validate the clinical utility of CEACAM1 in SARC.

In conclusion, our pan‐cancer and SARC‐focused analyses demonstrate that CEACAM1 is a robust biomarker associated with immune activation, favorable prognosis, and distinct therapeutic sensitivities. These findings suggest a promising role for CEACAM1 in guiding immunotherapeutic strategies and support its further investigation as a potential clinical biomarker and therapeutic target in SARC.

## Author Contributions

Conceptualization, Ziyou Lin, Yadong Guo, and Mengmei Zhang; methodology, Mengmei Zhang and Ziyou Lin; software, Ziqi Cao and Minjue Shan; validation, Ziqi Cao and Minjue Shan; formal analysis, Ziqi Cao and Minjue Shan; investigation, Ziqi Cao and Minjue Shan; resources, Mengmei Zhang and Yadong Guo; data curation, Ziqi Cao and Minjue Shan; writing–original draft, Ziqi Cao and Minjue Shan; writing–review and editing, Mengmei Zhang, Yadong Guo, and Ziyou Lin; visualization, Ziqi Cao and Minjue Shan; supervision, Mengmei Zhang, Yadong Guo, and Ziyou Lin; project administration, Mengmei Zhang, Yadong Guo, and Ziyou Lin.

## Funding

No funding was received for this manuscript.

## Disclosure

All authors have read and approved the final version of the manuscript.

## Ethics Statement

The authors have nothing to report.

## Conflicts of Interest

The authors declare no conflicts of interest.

## Data Availability

The datasets used and/or analyzed during the current study are available from the corresponding author on reasonable request.
